# Effect of Image Enhancement Filters on the Detection of Periapical Radiolucent Lesions on Panoramic Radiographs: A Multi-Reader Multi-Case Study

**DOI:** 10.3390/diagnostics16213460

**Published:** 2026-10-23

**Authors:** Ozlem Busra Dogan, Hatice Biltekin

**Affiliations:** Department of Dentomaxillofacial Radiology, Faculty of Dentistry, Bursa Uludağ University, Gorukle, Bursa 16120, Türkiye; haticebiltekin@uludag.edu.tr

**Keywords:** periapical diseases, image enhancement, image processing, computer-assisted, diagnosis, differential, dental students

## Abstract

**Background**: Panoramic radiography is widely used in dental practice, but its two-dimensional projection geometry and anatomical superimposition can obscure periapical radiolucent lesions, particularly in the maxillary anterior region and for novice observers. **Methods**: This retrospective multi-reader, multi-case study aimed to evaluate the effects of unsharp mask and Gaussian blur filters on the diagnostic performance and interobserver agreement of novice observers in detecting periapical radiolucent lesions on panoramic radiographs. Twenty final-year dental students evaluated 56 panoramic radiographs (28 lesion-positive and 28 lesion-free cases; 224 maxillary anterior teeth) under three imaging conditions (original, unsharp mask, and Gaussian blur). Observers scored lesion presence using a five-point confidence scale. Diagnostic performance (AUC, sensitivity, and specificity) was compared using the Dorfman–Berbaum–Metz/Obuchowski–Rockette multi-reader, multi-case method. Interobserver agreement was assessed using quadratic-weighted Cohen’s kappa and the intraclass correlation coefficient (ICC). **Results**: Imaging condition significantly affected AUC, sensitivity, and specificity (all *p* ≤ 0.003). Compared with original images, unsharp mask significantly improved sensitivity (78.2% vs. 52.5%), specificity (90.2% vs. 83.2%), and AUC (0.876 vs. 0.737) (all *p* < 0.05), with the highest interobserver agreement (ICC = 0.951; κ = 0.476). Gaussian blur significantly reduced sensitivity (40.2%) and AUC (0.655) compared with original images (both *p* < 0.001). **Conclusions**: Unsharp mask filtering was associated with improved diagnostic performance and interobserver agreement among novice observers, whereas Gaussian blur was associated with reduced lesion detection performance. These findings suggest that unsharp mask filtering may have potential as an adjunct to radiographic interpretation training.

## 1. Introduction

Panoramic radiography is one of the most frequently used imaging methods in dental practice and is widely used for a variety of diagnostic indications, including comprehensive dental examinations, orthodontic evaluations, implant planning, and the investigation of oral and maxillofacial disorders. Its relatively low radiation dose, wide anatomical coverage, and widespread availability make it a valuable first-line imaging technique in many clinical situations. The natural overlapping of anatomical structures in the two-dimensional projection geometry can mask pathological findings and reduce diagnostic accuracy in certain areas of the jaws. Periapical radiolucent lesions, often a reflection of apical periodontitis of endodontic origin, are among the most common pathological findings in the jaws; a global systematic review estimated the prevalence of apical periodontitis at the individual level to be 52% [1]. Since these lesions often develop and progress as a chronic, asymptomatic process [1], they are usually first encountered incidentally on radiographs obtained for unrelated purposes rather than for symptoms; periapical inflammatory lesions are among the incidental pathological findings reported on pre-treatment panoramic radiographs [2]. Therefore, timely radiographic diagnosis is a crucial factor for investigating and treating such lesions before they progress. The maxillary anterior region is one of the most challenging areas to interpret on panoramic radiographs. Anatomical superimposition from the nasal cavity, incisive canal, and anterior wall of the maxillary sinus can obscure subtle radiographic changes [3,4], making small or asymptomatic radiolucent lesions difficult to detect. 

Panoramic radiography has lower sensitivity than three-dimensional imaging for detecting periapical lesions, and its detection rate decreases as lesion size decreases because periapical radiolucencies may not become radiographically apparent until the adjacent cortical bone is involved [5,6]. Cone-beam computed tomography (CBCT) detects a substantial proportion of periapical lesions that are not visible on two-dimensional radiography [6,7]. In addition, the diagnostic performance of panoramic radiography for maxillary periapical radiolucencies is influenced by lesion size, anatomical location, and local contrast characteristics and AI-based detection studies [8,9,10,11].

Although imaging modalities such as periapical radiography and CBCT can improve diagnostic confidence [7,8], they also have practical limitations, including the need for additional examinations, increased cost, longer examination times, higher radiation exposure, and limited availability in routine clinical practice. Consequently, optimizing the diagnostic performance of panoramic radiography clinically important.

Panoramic radiography one of the most widely used imaging modalities in dental practice because of its broad diagnostic applicability, low radiation dose, and accessibility. Improving the conspicuity of periapical radiolucencies on panoramic radiographs may facilitate earlier lesion recognition and appropriate treatment planning. This need is particularly relevant for novice observers, who are more likely to overlook subtle or early-stage lesions because of the complex anatomical background of the maxillary anterior region. Digital image-processing techniques offer a practical approach to enhancing lesion conspicuity while preserving the original image data. Filtering methods alter the spatial frequency content of an image and can thereby change the visibility of anatomical structures and pathological findings. Edge-enhancement filters such as unsharp mask emphasize high-frequency components and may increase the conspicuity of subtle findings, whereas Gaussian blur suppresses high-frequency information and softens the image, potentially impairing lesion visualization [12].

Most previous evaluations have been performed in vitro or ex vivo using extracted teeth, dry skulls, or simulated lesions [13,14,15,16,17,18,19]. These models are useful for assessing the physical effects of filtering on image quality and spatial resolution, but they do not reproduce the anatomical overlaps, positional errors, and noise characteristic of clinical panoramic imaging. Consequently, how these filters perform diagnostically in vivo is not well established. In addition, most current studies deal with intraoral radiography rather than panoramic imaging [13,19,20,21], and the effect of image enhancement, particularly on the diagnostic performance of novice observers and inter-observer agreement, has received little attention.

The aim of this study was to investigate the impact of two digital image-processing filters (unsharp mask and Gaussian blur) on the diagnostic performance and interobserver agreement of novice observers when detecting periapical radiolucent lesions in the maxillary anterior region on panoramic radiographs. We hypothesized that unsharp mask filtering would improve diagnostic performance and interobserver agreement by enhancing lesion conspicuity through increased edge definition and local contrast, whereas Gaussian blur filtering would reduce diagnostic performance by attenuating fine trabecular detail and obscuring lesion boundaries. Given the greater diagnostic uncertainty experienced by novice observers, we further hypothesized that effective image enhancement could improve not only diagnostic performance but also the consistency of radiographic interpretation, with potential value for radiology training.

## 2. Methods

The study was approved by the Health Research Ethics Committee of Bursa Uludağ University (Approval No: 2025/989/21-4) and conducted in accordance with the Declaration of Helsinki and its later amendments. Written informed consent was obtained from all participants involved in observer calibration and reliability procedures. Because the study used anonymized images originally acquired for routine diagnostic purposes, the Ethics Committee waived the requirement for individual informed consent for image use. Furthermore, all patients treated at our institution routinely provide written general consent allowing the use of their anonymized clinical and radiographic data for research purposes. Bursa Uludağ University Faculty of Dentistry provides services at Bursa Training and Research Hospital.

**Figure 1:**
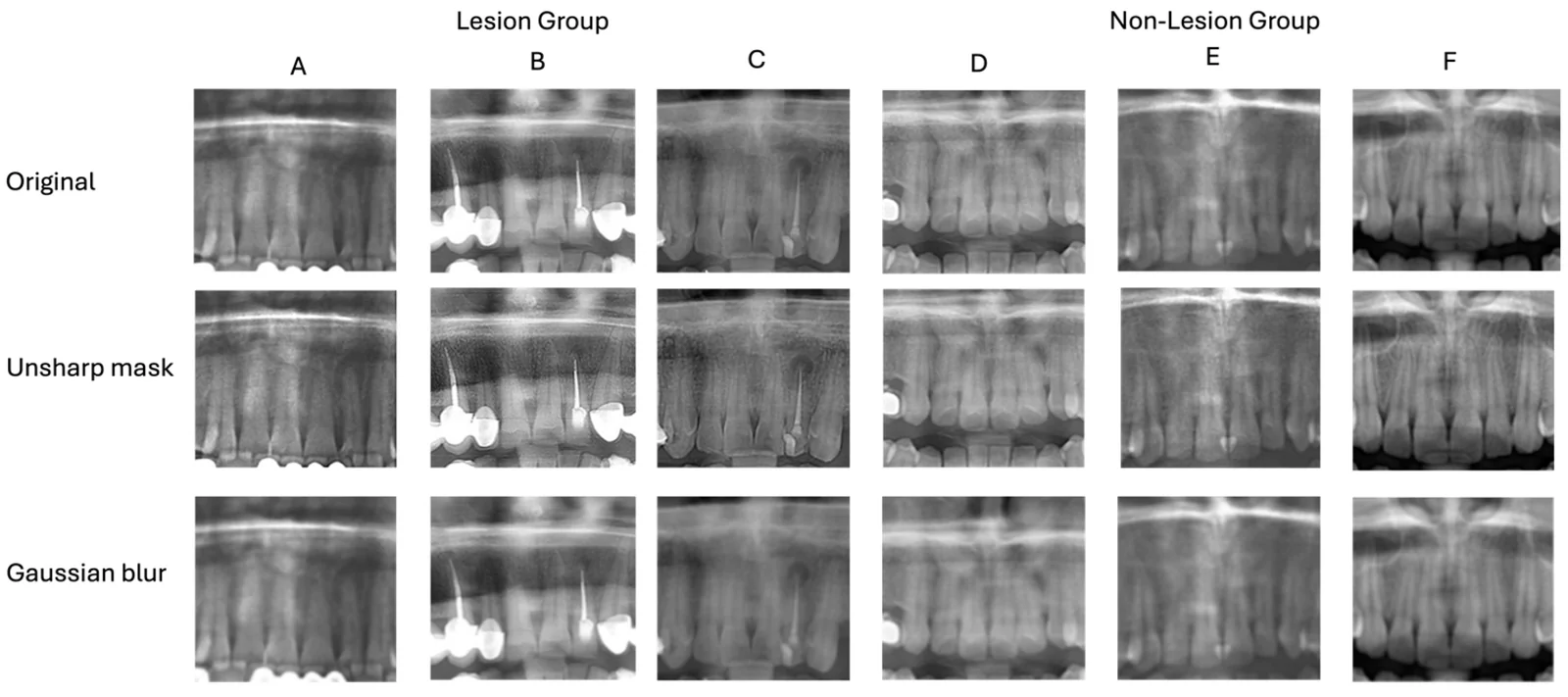
Representative cropped panoramic radiograph panels demonstrating the lesion group (**A**–**C**) and non-lesion group (**D**–**F**) across the three evaluated imaging conditions: original images (**top row**), unsharp mask filtering (**middle row**), and Gaussian blurring (**bottom row**). Note: During observer evaluations, panoramic radiographs were displayed in their full, uncropped format. Cropped images are presented here solely to provide detailed visualization of the maxillary anterior region (FDI tooth numbers 12, 11, 21, and 22).

This single-center, retrospective diagnostic accuracy study used a multi-reader, multi-case (MRMC) design to examine how digital image processing filters affect detection of periapical radiolucent lesions in the maxillary anterior region on panoramic radiographs. Each panoramic radiograph was evaluated under three conditions—original, unsharp mask, and Gaussian blur—in a within-subject, repeated-measures arrangement (Figure 1) so that each case served as its own control.

### 2.1. Case Selection and Region of Interest

Analysis was restricted to the maxillary anterior region (#11, #12, #21, and #22) to preserve anatomical homogeneity. Twenty final-year dental students who had completed theoretical and clinical training in oral and maxillofacial radiology served as novice observers.

CBCT was used as the reference standard to determine the presence or absence of periapical lesions at the tooth level. CBCT images had been acquired for various clinical indications within 30 days before the panoramic radiography using a Planmeca ProMax 3D (Planmeca Oy, Helsinki, Finland) device. Patients with available CBCT examinations in the clinical archive were screened for eligibility. Two oral and maxillofacial radiologists with at least four years of experience independently evaluated the CBCT images to identify periapical lesions, defined as hypodense areas at the root apex visible in at least two planes.

Disagreements between the two radiologists were resolved by consensus discussion. The same radiologists also assessed the diagnostic acceptability of the corresponding panoramic radiographs, and cases were included based on their consensus. Lesion status was determined solely from the CBCT images; the panoramic radiographs were used only to assess image quality, and no student reading contributed to the reference standard. The reference standard was therefore independent of the index test, which minimized the risk of incorporation bias. The student observers evaluated only the panoramic radiographs and were blinded to the CBCT findings and the radiologists’ assessments. Histopathological confirmation was unavailable because biopsies were not routinely performed for these lesions.

Lesion-positive cases were included when a periapical radiolucent lesion involving tooth #12, #11, #21, or #22 was confirmed on CBCT and the corresponding panoramic radiograph was of diagnostically acceptable quality. Lesions of varying sizes were included. Cases in which a single lesion extended over the apices of two or more adjacent teeth were excluded so that each lesion could be attributed to one tooth. A case could contain more than one lesion-positive tooth if the lesions were separate.

The absence of lesions was determined using existing CBCT scans available in the institutional imaging archive, obtained within 30 days of the corresponding panoramic examination. Controls were selected to represent realistic non-pathological presentations, including normal tooth structure, restored or root-treated teeth without periapical pathology, mild periodontal bone loss, anatomical structures such as the incisive canal or nasal floor, and minor technical or positioning artifacts commonly encountered on panoramic radiographs.

### 2.2. Sample Size Estimation

Sample size was estimated a priori, before data collection began, at the panoramic image level using G*Power (Version 3.1.9.7; Heinrich-Heine-Universität Düsseldorf, Germany) since teeth within the same radiograph could not be treated as statistically independent observations. Because no directly comparable prior study existed for unsharp mask/Gaussian blur filtering of panoramic images in the maxillary anterior region, the calculation drew on effect sizes reported in related observer-performance studies of digital image processing and diagnostic accuracy in dental radiology; a moderate effect size (Cohen’s f = 0.25) was assumed accordingly, with α = 0.05 and power (1 − β) = 0.80 for a within-subject, repeated-measures design across three imaging conditions [20]. This yielded a minimum requirement of 54 panoramic radiographs; 56 were included to allow for possible exclusions, comprising 28 lesion-positive and 28 lesion-free cases. In lesion-positive cases, at least one maxillary anterior tooth showed a radiolucent lesion on CBCT, while the remaining evaluated teeth in that case could be lesion-free. Observer assessments were made at the tooth level for each of the four maxillary anterior teeth, yielding 224 tooth-level observations per imaging condition (672 tooth × condition evaluations per observer).

### 2.3. Observers and Image Evaluation

Twenty final-year dental students who had completed formal training in oral and maxillofacial radiology served as observers. They were selected to represent less-experienced clinicians so that the effect of image processing filters could be assessed in a novice population. Prior to the main study, a feasibility pilot study involving four final-year dental students evaluating 15 panoramic radiographs in a single session was conducted to optimize viewing conditions, session duration, and the image-presentation workflow. Diagnostic performance was not assessed at this stage. Observers reported their subjective fatigue on a 5-point Likert scale (1 = not fatigued, 5 = extremely fatigued), and feedback regarding usability and the clarity of the evaluation criteria was recorded. Data from the pilot assessment were not included in the final analysis.

Before the main reading sessions, all observers attended a brief calibration session to standardize the evaluation procedure and to familiarize them with the 5-point confidence scale. Because all observers had already completed formal training in oral radiology, no additional theoretical instruction on lesion characteristics was provided, and no information about the presence or type of image filters was given.

All evaluations were performed on the same 15-inch laptop featuring a display resolution of 2880 × 1864 pixels (224 ppi) at a viewing distance of approximately 50 cm under dim ambient lighting and with constant display settings. Zoom, brightness, and contrast adjustments were disabled, and file names were anonymized to prevent identification of the filter type.

Observers were blinded to case diagnoses and reference-standard findings and were not told which filter was applied; however, differences in image appearance between conditions were inevitably perceptible. The two oral and maxillofacial radiologists who established the reference standard did not rate the images in the reading sessions. All reading sessions were supervised by one of these two radiologists, who ensured adherence to the reading protocol (display settings, image order, and session timing) and did not comment on the images or influence the observers’ ratings. The students had no access to the CBCT findings or to the radiologists’ assessments at any time.To reduce recall bias, each observer completed three reading sessions, each containing all 56 radiographs under a single imaging condition (168 radiographs per observer overall). Evaluation rounds were separated by a washout period of at least seven days, an interval comparable to that used in previous observer-performance studies [22,23,24]. The order of imaging conditions was randomized across observers, and within each session, image presentation order was randomized independently for each observer using a computer-generated sequence so that no two observers viewed the images in the same sequence, and no case appeared in more than one version within a single session. Observers had no access to their previous ratings. Over the course of the study, each observer rated every case under all three imaging conditions. Splitting the evaluation into three sessions was intended to limit observer fatigue.

Each radiograph was rated on a 5-point confidence scale ranging from “definitely absent” to “definitely present” (1 = definitely no lesion, 2 = probably no lesion, 3 = uncertain, 4 = probably lesion present, 5 = definitely lesion present). For the primary analysis, these scores were dichotomized, with 1–3 as negative and 4–5 as positive, a threshold chosen to reflect a conservative clinical stance in which uncertainty counts as a negative finding. The full 5-point scale was retained separately for ROC analysis, AUC calculation, and agreement analysis, providing a threshold-independent measure of diagnostic performance.

### 2.4. Digital Image Processing 

Panoramic radiographs were retrieved retrospectively from the institutional archive. Images had been acquired with a Veraview IC5 HD panoramic imaging system (Morita Corporation, Osaka, Japan) using standardized exposure parameters (1–7.5 mA, 60–70 kV, 5.5–10 s) and exported in TIFF format. All images were converted to 8-bit grayscale before processing. Processing was performed in Fiji/ImageJ (version 1.54p; National Institutes of Health, Bethesda, MD, USA) using automated batch processing with identical settings for all images to avoid operator bias, producing three versions of each case: unprocessed original, unsharp mask-filtered, and Gaussian blur-filtered [12]. Both filters were applied to the original image. Filter parameters were specified in pixel units.

Unsharp masking (USM) enhances edge contrast by emphasizing high-frequency components. The technique subtracts a Gaussian-blurred version of an image from the original and adds the resulting high-frequency information back:

*I_enhanced_* = *I_original_* + *k*(*I_original_* − *Gaussian*(*I_original_*))

where *I_original_* is the original image, *Gaussian*(*I_original_*) its Gaussian-blurred counterpart, and k the enhancement coefficient (scaling factor). A radius (Gaussian standard deviation, σ) of 2 pixels and a mask weight (*w*) of 0.6 were used throughout. In ImageJ, unsharp masking is implemented as (I_original − *w* Gaussian(I_original))/(1 − *w*), which is mathematically equivalent to the equation above with k = *w*/(1 − *w*). A mask weight of 0.6 therefore corresponds to a scaling factor of k = 1.5.

Gaussian blur, applied separately to all images, acts as a low-pass filter that smooths high-frequency components and reduces noise:

$$G\left(x,y\right)=\frac{1}{2\pi \sigma^{2}}e^{-\frac{x^{2}+y^{2}}{2\sigma^{2}}}$$

where *x* and *y* are spatial coordinates and σ the standard deviation governing blur strength. A σ value of 2 pixels was used across all images. In ImageJ, the kernel size is not set manually but is derived automatically from σ; for σ = 2 pixels on 8-bit images this results in a kernel radius of approximately 9 pixels (approximately 19 × 19 pixels).

These parameter values (unsharp mask radius/mask weight and Gaussian blur σ) were selected by consensus between two oral and maxillofacial radiologists following visual assessment during the pilot study, with the aim of achieving a moderate, clinically plausible degree of sharpening or blurring without introducing obvious image artifacts while remaining consistent with the parameter ranges reported in previous dental radiographic filtering studies [15,17].

### 2.5. Statistical Analysis

The tooth served as the unit of analysis, with lesion presence or absence assessed separately for teeth #12, #11, #21, and #22. Because each observer evaluated every tooth under all three imaging conditions, the data comprised repeated measurements clustered within both observers and cases. Accordingly, formal comparisons were performed using a multi-reader, multi-case (MRMC) approach.

Descriptive diagnostic performance metrics, including sensitivity, specificity, positive predictive value (PPV), and negative predictive value (NPV), were calculated for each imaging condition from the dichotomized scores (1–3 = negative, 4–5 = positive) and are reported with Wilson score confidence intervals.

Formal comparisons of AUC, sensitivity, and specificity across imaging conditions were performed using the Dorfman–Berbaum–Metz (DBM) jackknife method within the Obuchowski–Rockette (OR) multi-reader, multi-case (MRMC) framework [25,26], the standard approach for MRMC diagnostic accuracy studies. In this framework, both readers and cases were modeled as crossed random effects, whereas imaging condition was treated as a fixed effect. For each combination of imaging condition and observer, a figure of merit was calculated (AUC from the 5-point confidence ratings; sensitivity and specificity from the dichotomized scores). Case-level jackknife pseudovalues were generated by omitting each of the 56 cases in turn and recalculating the corresponding figure of merit. The resulting imaging condition × reader × case pseudovalue array was analyzed using a three-way analysis of variance. The overall effect of imaging condition was evaluated using the DBM F-statistic, with denominator degrees of freedom estimated using the Satterthwaite approximation. Pairwise comparisons between imaging conditions were subsequently performed to estimate mean differences, 95% confidence intervals, and corresponding *p*-values.

Interobserver agreement was assessed using quadratic-weighted Cohen’s kappa [27], averaged across all 190 possible observer pairs, an agreement measure appropriate for ordinal rating scales. Uncertainty around the mean weighted kappa and between-condition differences was estimated using a case-level cluster bootstrap with 300 resamples, which preserves the correlation of ratings belonging to the same case, and 95% confidence intervals were derived from the bootstrap distribution. For comparability with previous imaging studies, the two-way random-effects intraclass correlation coefficient (ICC) based on absolute agreement was also calculated using the 5-point confidence ratings and reported as the average-measures coefficient, ICC(2,20).

Descriptive statistics were computed in IBM SPSS Statistics (IBM Corp., Armonk, NY, USA). DBM/OR jackknife analyses and agreement statistics were performed in R (version 4.6.1; R Foundation for Statistical Computing, Vienna, Austria). A two-sided *p*-value < 0.05 was considered statistically significant.

## 3. Results

Twenty final-year dental students evaluated 224 maxillary anterior teeth (#11, #12, #21, #22) on 56 panoramic radiographs under three imaging conditions (original, unsharp mask, and Gaussian blur).

### 3.1. Interobserver Agreement

**Figure 2:**
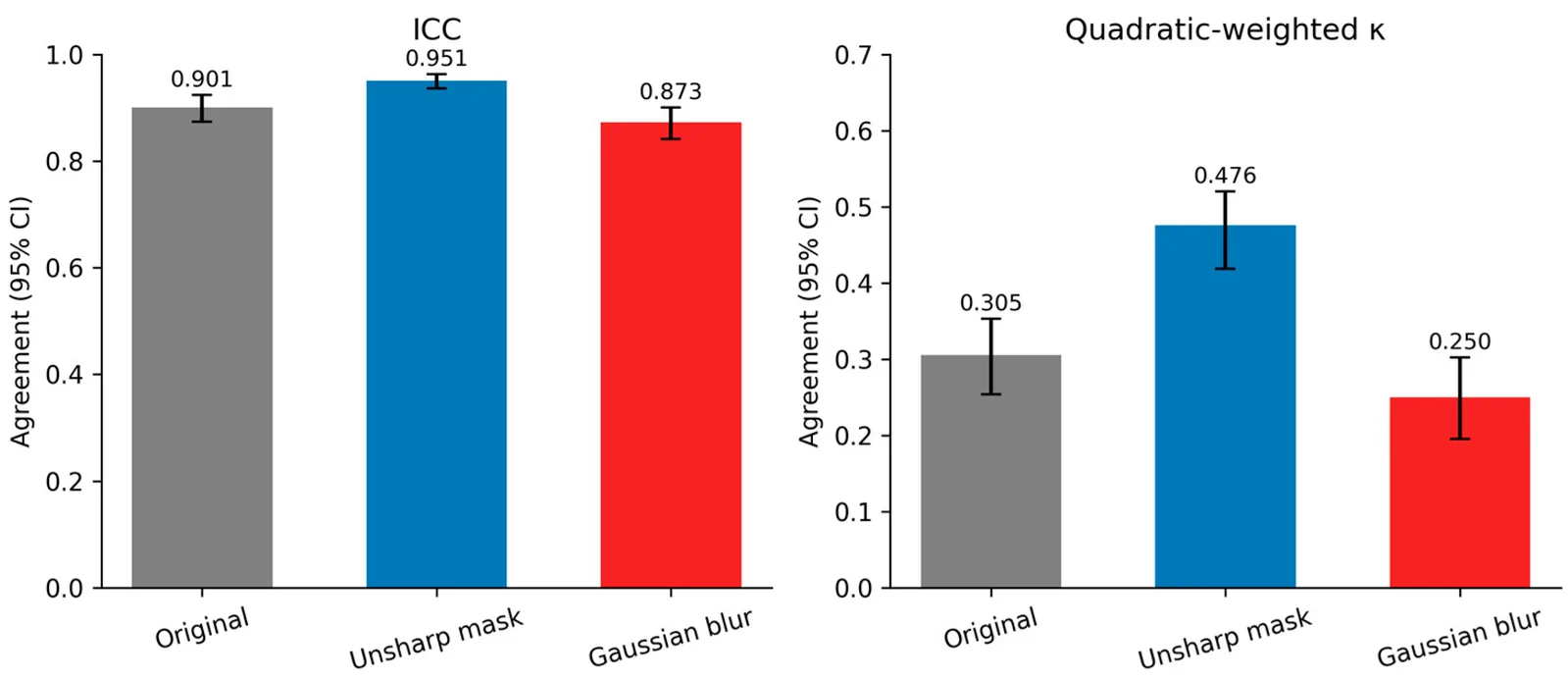
Interobserver agreement under each imaging condition, expressed as (**left**) the intraclass correlation coefficient (ICC) and (**right**) the mean pairwise quadratic-weighted Cohen’s κ across all 190 observer pairs. Error bars indicate 95% confidence intervals (κ: 300-resample case-level cluster bootstrap).

Table 1 and Figure 2 summarize interobserver agreement under each imaging condition using both the ICC and quadratic-weighted kappa. The two indices produced a broadly consistent ranking—unsharp mask highest, Gaussian blur lowest—but differed considerably in absolute magnitude. ICC values (average-measures, reflecting the reliability of the mean rating of the 20 observers) suggested excellent agreement across all three conditions (0.873–0.951), whereas weighted kappa, which reflects agreement between individual observer pairs and is more appropriate for the ordinal five-point scale used here, indicated only fair-to-moderate agreement (0.250–0.476).

The improvement in agreement with unsharp mask relative to the original images was statistically significant (Δκ = 0.171, 95% CI 0.089–0.241, *p* < 0.001), whereas the apparent reduction in agreement with Gaussian blur was not (Δκ = −0.055, 95% CI −0.126–0.015, *p* = 0.133).

Mean pairwise quadratic-weighted Cohen’s κ across all 190 observer pairs. The 95% confidence intervals were estimated using a 300-resample case-level cluster bootstrap.

### 3.2. Diagnostic Accuracy: AUC, Sensitivity, and Specificity

Omnibus DBM/OR jackknife tests indicated a statistically significant overall effect of imaging condition on all three diagnostic performance metrics: AUC (F(2,68.1) = 26.9, *p* < 0.0001), sensitivity (F(2,69.6) = 41.7, *p* < 0.0001), and specificity (F(2,52.3) = 6.5, *p* = 0.003).

**Figure 3:**
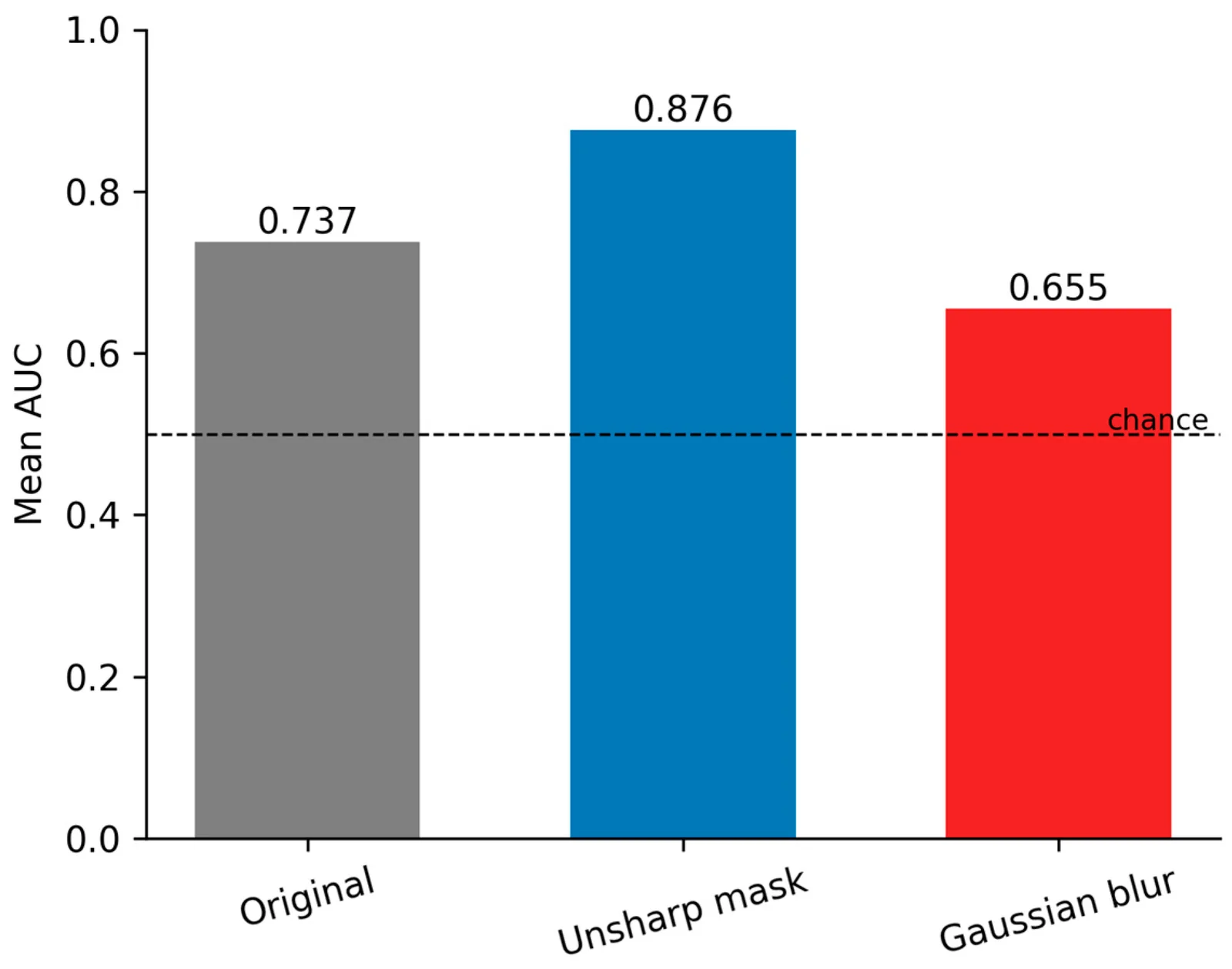
Mean AUC by imaging condition (mean of per-observer jackknife figures of merit). The dashed line indicates chance-level discrimination (AUC = 0.5).

**Figure 4:**
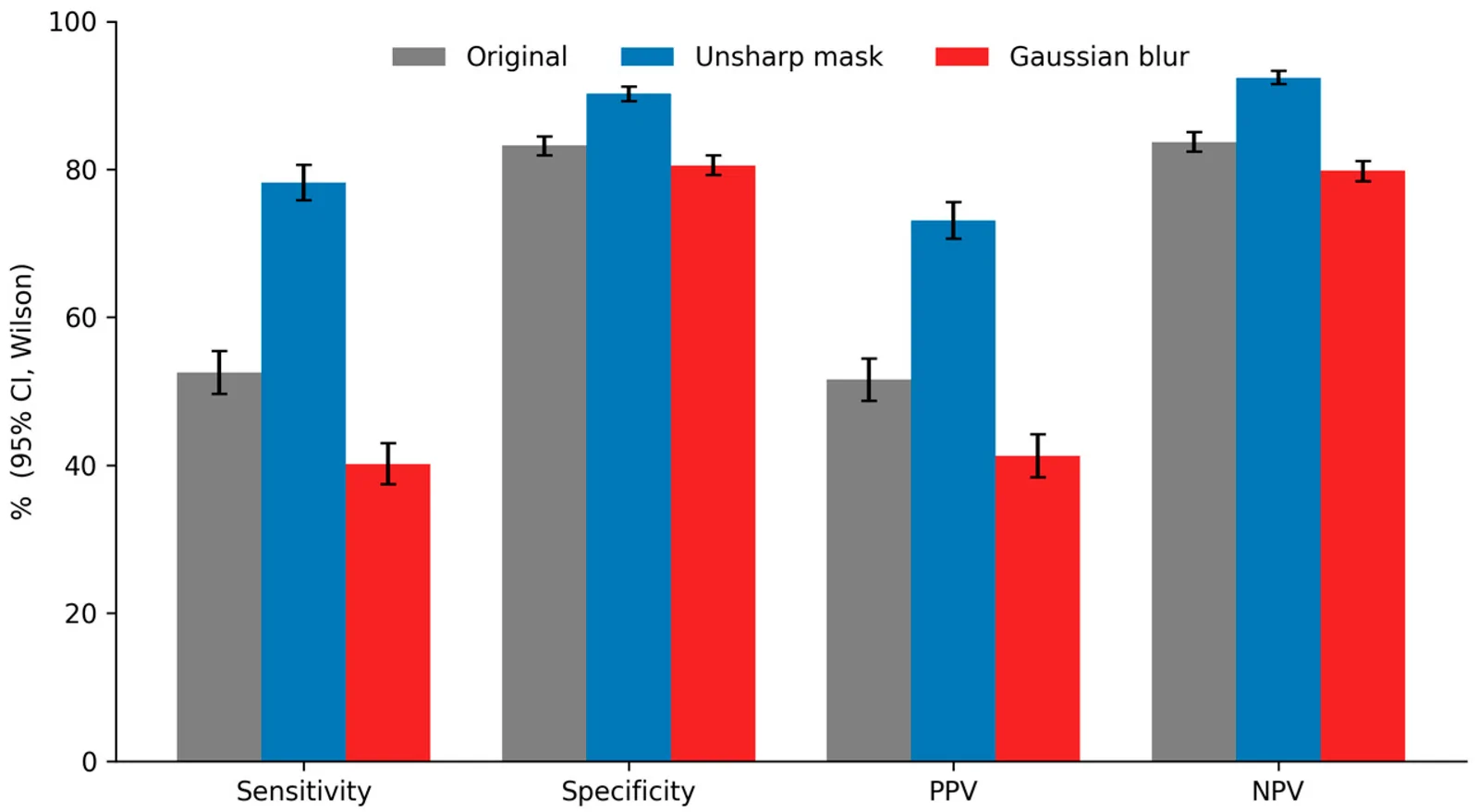
Sensitivity, specificity, positive predictive value (PPV), and negative predictive value (NPV) by imaging condition, calculated at the tooth level from dichotomized observer responses. Error bars indicate 95% Wilson score confidence intervals (descriptive; not adjusted for clustering of ratings within observers and cases).

Table 2 and Figure 3 summarize the mean AUC by condition. Table 3 and Figure 4 summarize sensitivity, specificity, PPV, and NPV derived from the dichotomized responses, which were calculated at the tooth level; confidence intervals were estimated using the Wilson score method. Table 4 presents the formal DBM/OR pairwise comparisons underlying the significance statements in this section.

**Figure 5:**
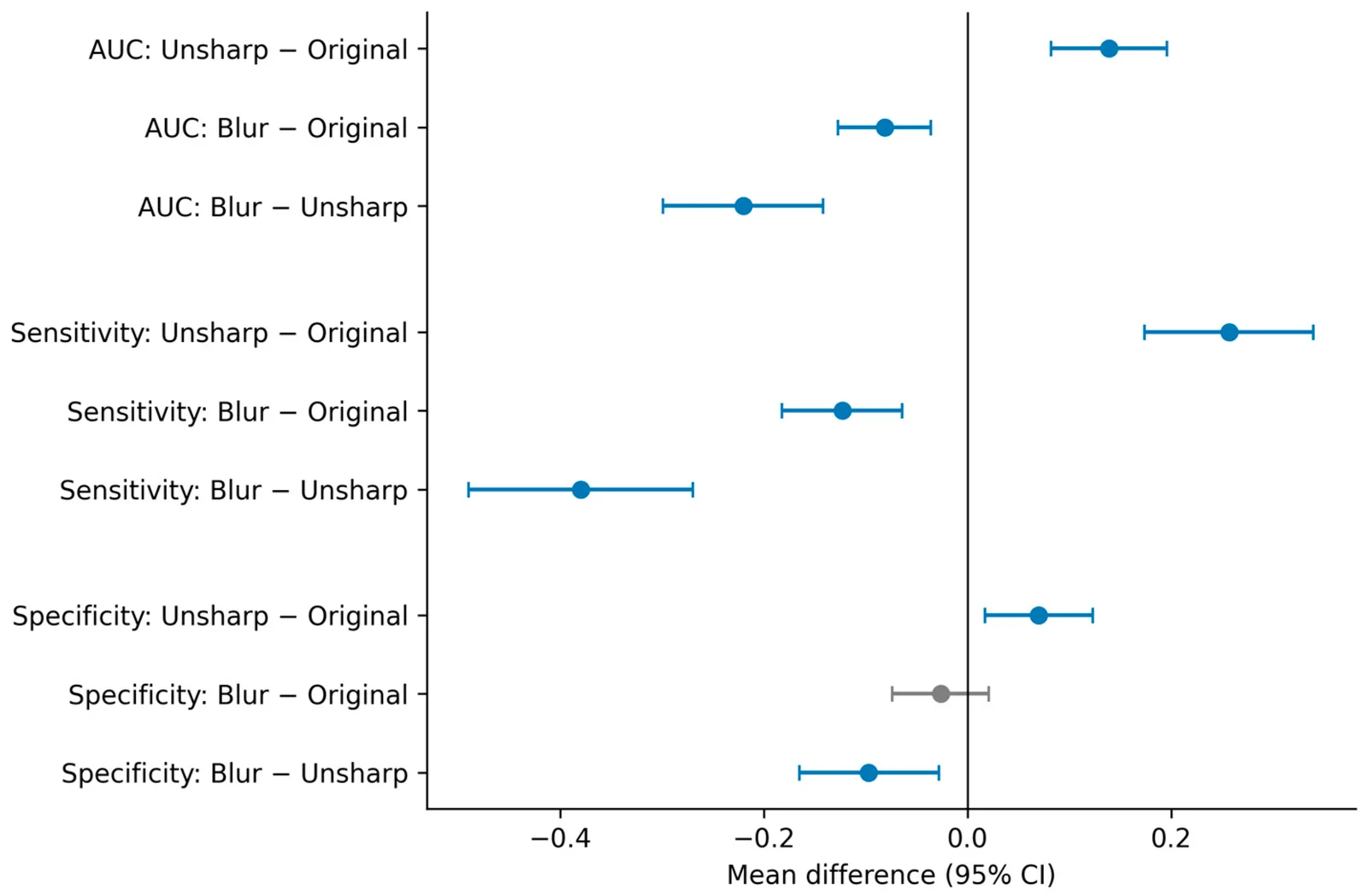
Forest plot of DBM/OR jackknife pairwise differences in AUC, sensitivity, and specificity between imaging conditions. Horizontal bars indicate 95% confidence intervals; comparisons whose intervals include zero are shown in grey.

Compared with the original images, unsharp mask filtering significantly increased AUC, sensitivity, and specificity (Table 4, Figure 5). In contrast, Gaussian blur significantly reduced AUC and sensitivity, whereas the reduction in specificity was not statistically significant (Table 4, Figure 5).

## 4. Discussion

This study evaluated the effects of two digital image-processing filters (unsharp mask and Gaussian blur) on the detection of apical radiolucent lesions in the maxillary anterior region on panoramic radiographs by final-year dental students. Unsharp mask filtering significantly improved sensitivity, specificity, AUC, and interobserver agreement compared with the original images. In contrast, Gaussian blur reduced sensitivity, and AUC but had no significant effect on specificity or interobserver agreement. Notably, unsharp mask filtering also outperformed Gaussian blur across all three metrics, including a significant advantage in specificity, suggesting that the two filters affected novice observers’ diagnostic performance in opposite directions. These findings suggest that Gaussian blur mainly impaired the detection of true lesions rather than increasing false-positive findings in lesion-free teeth. Because panoramic radiography is widely used for dental assessment, improving lesion detection at this stage may support earlier diagnosis and appropriate treatment planning, particularly for novice observers.

The beneficial effect of the unsharp mask filter is likely due to improved edge definition and local contrast, which increases the visibility of the lesion against the anatomically complex background of the maxillary anterior region. This effect may be particularly valuable for novice observers who are more likely to overlook radiographic findings due to anatomical overlap and limited interpretation experience. These findings are consistent with previous studies showing that sharpening filters improves the visibility of periapical radiolucencies and other small-bone abnormalities. Previous studies have reported higher ROC/AUC values for sharpened periapical radiographs, especially when evaluating subtle bone changes and vertical root fractures [17,28].

Because intraoral imaging systems such as photostimulable phosphor plate sensors inherently provide high spatial resolution, digital image enhancement filters may provide only limited additional diagnostic benefit. An in vitro study using periapical radiographs reported that although image filtering improved perceived image quality, it did not significantly improve diagnostic accuracy [13]. A CBCT study reported that sharpening filters improved diagnostic performance only under certain imaging conditions, such as reduced radiation dose (low tube current), indicating that the effectiveness of image enhancement depends on the imaging situation rather than representing a universal benefit [28].

Although our findings are based on an image processing approach that does not involve artificial intelligence, they also align with a growing literature evaluating AI-based decision support for periapical lesion detection. Hamdan et al. reported that a deep learning tool improved the sensitivity and specificity of general dentists in detecting apical radiolucencies in periapical radiographs by 8.2% and 23.1%, respectively [29], and a later study by the same author found no statistically significant improvement when the same tool assisted oral and maxillofacial radiologists [30]. Liu et al., whose findings are more directly comparable to ours, showed that deep-learning model support increased the mean AUC of novice dentists interpreting periapical radiographs from 0.88 to 0.94 [31]—an improvement in the same direction to the AUC gain we observed with sharpening mask filtering alone (from 0.737 to 0.876) achieved without any AI component. Taken together, these findings suggest that at least some of the reported diagnostic benefit for AI-based tools in this field can be achieved or supplemented through relatively simple and widely accessible image enhancement techniques; this may be particularly important for training or practice settings that lack easy access to AI-based diagnostic support.

The reduction in sensitivity associated with Gaussian blur in the present study fits expectations from the literature, where noise-reducing filters have been reported to increase measurement error by smoothing away fine detail [12,32]. Our MRMC analysis indicates that the detrimental effect of Gaussian blur was primarily observed in the detection of true lesions, whereas its effects on false-positive findings in lesion-free teeth and on interobserver consistency were comparatively limited. This distinction would have been difficult to establish using analyses that treat tooth-level observations as independent. The sizeable sensitivity gain observed here—from 52% with original images to 78% with sharpened ones—is worth interpreting with some caution since it likely reflects the observer profile and the conservative scoring threshold used in this analysis as much as the filter itself. 

Our observers were senior dental students with limited experience in oral and maxillofacial radiology. For sensitivity and specificity analyses, confidence scores of 1–3 were classified as negative because they did not represent a definitive positive diagnosis. Many lesions received indeterminate gradings on the original panoramic images, possibly causing relatively low sensitivity. Sensitivity increased significantly after unsharp mask filtering. Sharpening may help novice observers recognize radiolucent lesions with greater confidence by making lesion edges more distinct. In clinical practice, a suspicious finding often requires additional imaging such as periapical radiography or CBCT. However, for novice observers, uncertainty may also reflect limited interpretive experience; this suggests that image enhancement may be particularly useful during radiography training. The increase in positive ratings following unsharp masking should be interpreted as an improvement in the performance of novice observers and not as evidence that image enhancement can replace additional imaging when clinically indicated. These findings suggest that image enhancement may have value as an educational tool to increase confidence and consistency during radiographic interpretation, but this possibility requires further investigation.

The difference between the ICC and quadratic-weighted kappa also warrants consideration. Although the ICC indicated excellent agreement across all three imaging conditions (0.87–0.95), it treats the five-point confidence scale as a continuous variable and primarily reflects consistency in the relative ranking of cases. In contrast, quadratic-weighted kappa, which is better suited to ordinal rating scales, indicated only fair-to-moderate agreement (0.25–0.48) [33]. Notably, unsharp masking significantly improved weighted kappa, whereas no significant difference was observed between the original and Gaussian-blurred images. The weighted kappa values observed in the present study are also consistent with previous research on digitally filtered panoramic radiographs. Baksi et al. likewise reported fair-to-moderate interobserver agreement using Cohen’s kappa when evaluating anatomical structures on filtered panoramic images [24]. Together, these findings suggest that, for ordinal confidence ratings, kappa-based measures may provide a more appropriate measure of observer agreement for this task than the substantially higher ICC values. This interpretation is further supported by methodological studies showing that ICC and quadratic-weighted kappa, although related, may diverge considerably when applied to ordinal rating data, particularly when raters differ in their use of the rating scale [34]. These findings highlight that, in observer-performance studies using ordinal confidence scales, reliance on the ICC alone may overestimate interobserver agreement. Quadratic-weighted kappa, or other agreement measures appropriate for ordinal data, should therefore be reported alongside the ICC.

This gap reflects the differing mathematical properties of the two indices. The ICC is a variance-based measure that evaluates relative consistency across raters and increases when average-measures forms are used, as random disagreements cancel out. In contrast, quadratic-weighted kappa quantifies agreement between individual observer pairs after adjusting for chance agreement based on each observer’s rating distribution. Because most teeth in this study were lesion-free and were therefore likely to receive low confidence ratings on the five-point scale, substantial chance agreement further lowered the kappa values. Thus, while observers demonstrated high overall consistency in ranking cases, they frequently differed on exact categorical ratings, making quadratic-weighted kappa a more conservative and clinically relevant metric for individual reader agreement.

Several limitations of this study should be acknowledged. First, regarding observer experience, all observers were final-year dental students representing novice readers; the benefits of unsharp masking observed here may not directly apply to experienced radiologists. In experienced radiologists, who rely more on internalized anatomical knowledge, the same enhancement may be less beneficial and could even increase false-positive findings because halo and edge artifacts around high-contrast structures (e.g., root outlines, restorations, and the nasal floor) may be misinterpreted as pathology. Our results should therefore not be extrapolated to experienced clinicians, and the effect of unsharp masking on false-positive rates in these readers remains to be tested. Further studies involving clinicians with different levels of experience are needed to better understand the diagnostic benefit of unsharp masking. Second, the reference standard was CBCT without histopathological confirmation; although CBCT is considerably more accurate than two-dimensional imaging, it remains an imperfect reference standard for periapical pathology, and some misclassification of lesion status cannot be excluded. Third, the sample size was relatively small, consisting of panoramic radiographs evaluated in a single center. Although sample size was calculated a priori and the fully crossed multi-reader multi-case (MRMC) design increased statistical efficiency, the limited number of patients and lesions reduces estimate precision and may not capture the full variability of periapical lesions in terms of size, location, and anatomical background. Fourth, regarding image acquisition and filter configuration, observers worked with static images without dynamic adjustments (such as manual brightness/contrast changes or free zooming) to isolate the filter effects, and only a single parameter set was tested for each filter, selected by consensus rather than systematic optimization; consequently, alternative display conditions or filter settings may yield different results. Because the effect of such filters depends on image resolution, noise characteristics, and exposure settings, our findings are specific to the Veraview IC5 HD system and the processing pipeline used here. Multi-center studies using different imaging systems and systematic parameter optimization are needed to confirm generalizability. Fifth, regarding imaging and display, evaluations were restricted to panoramic radiographs of the maxillary anterior region and performed on standardized laptop displays rather than medical-grade diagnostic monitors. Posterior regions show heavier superimposition of anatomical structures (e.g., the zygomatic buttress and maxillary sinus floor), which may alter both the benefit of edge enhancement and the risk of artifacts; our results should therefore not be generalized to posterior areas. This choice is noteworthy because the diagnostic impact of image enhancement filters varies considerably across different imaging modalities (including intraoral, extraoral, and handheld X-ray systems), anatomical sites, and diagnostic tasks, such as root resorption, caries detection, and peri-implant bone assessment [35,36,37]. Consequently, how these filters perform in posterior regions under interactive display conditions or for radiopaque and mixed lesions remains an open question. Sixth, although a washout period of at least seven days, independent randomization of image order, and blinding were implemented, residual recall bias cannot be fully excluded. Finally, regarding agreement metrics, the five-point confidence scale was ordinal, and because the ICC and weighted κ capture distinct mathematical aspects of agreement, observer agreement on exact categories remained only fair to moderate despite high ICC values.

## 5. Conclusions

Unsharp mask filtering was associated with higher diagnostic performance and interobserver agreement among novice observers for detecting apical radiolucent lesions on panoramic radiographs, whereas Gaussian blur was associated with reduced sensitivity. These findings suggest that edge-enhancement filtering may have potential as an image-processing aid in dental education. Further multicenter studies including experienced observers are warranted to assess the generalizability of these findings across different clinical settings.

## Figures and Tables

**Table 1:** Interobserver agreement under different image-processing conditions.

Filter Condition	ICC(2,20), Average-Measures (95% CI)	Quadratic-Weighted κ (95% CI) ^a^
Original	0.901 (0.874–0.924)	0.305 (0.254–0.353)
Unsharp Mask	0.951 (0.936–0.963)	0.476 (0.419–0.520)
Gaussian Blur	0.873 (0.841–0.901)	0.250 (0.195–0.302)

ICC, intraclass correlation coefficient (two-way random effects, absolute agreement); CI, confidence interval. ^a^ Mean of pairwise quadratic-weighted Cohen’s κ across all 190 observer pairs; 95% CIs from a 300-resample case-level cluster bootstrap. The improvement in κ with unsharp mask relative to the original images was significant (Δκ = 0.171, 95% CI 0.089–0.241, *p* < 0.001); the reduction with Gaussian blur was not (Δκ = −0.055, 95% CI −0.126–0.015, *p* = 0.133).

**Table 2:** Mean AUC by imaging condition derived from the DBM/OR jackknife analysis.

	Mean AUC
Original	0.737
Unsharp Mask	0.876
Gaussian Blur	0.655

AUC, area under the receiver operating characteristic curve.

**Table 3:** Diagnostic performance metrics based on dichotomized observer responses.

Imaging Condition	Sensitivity % (95% CI)	Specificity % (95% CI)	PPV % (95% CI)	NPV % (95% CI)
Original	52.5 (49.6–55.4)	83.2 (81.9–84.4)	51.6 (48.7–54.4)	83.7 (82.4–85.0)
Unsharp Mask	78.2 (75.8–80.6)	90.2 (89.2–91.2)	73.1 (70.6–75.6)	92.4 (91.5–93.3)
Gaussian Blur	40.2 (37.4–43.0)	80.5 (79.2–81.9)	41.3 (38.4–44.2)	79.8 (78.4–81.1)

PPV, positive predictive value; NPV, negative predictive value; CI, confidence interval.

**Table 4:** Dorfman–Berbaum–Metz/Obuchowski–Rockette (DBM/OR) jackknife pairwise comparisons of diagnostic performance across imaging conditions.

Metric	Comparison	Δ	95% CI	*p*
AUC	Unsharp Mask—Original	+0.139	0.082 to 0.196	<0.001
AUC	Gaussian Blur—Original	−0.081	−0.127 to −0.036	<0.001
AUC	Gaussian Blur—Unsharp Mask	−0.220	−0.299 to −0.142	<0.001
Sensitivity	Unsharp Mask—Original	+0.257	0.174 to 0.340	<0.001
Sensitivity	Gaussian Blur—Original	−0.123	−0.182 to −0.064	<0.001
Sensitivity	Gaussian Blur—Unsharp Mask	−0.380	−0.490 to −0.270	<0.001
Specificity	Unsharp Mask—Original	+0.070	0.017 to 0.123	0.012
Specificity	Gaussian Blur—Original	−0.026	−0.074 to 0.021	0.262
Specificity	Gaussian Blur—Unsharp Mask	−0.097	−0.165 to −0.028	0.008

AUC, area under the receiver operating characteristic curve; CI, confidence interval; Δ, the mean difference in the jackknife figure of merit (AUC for AUC comparisons and proportion for sensitivity and specificity comparisons). Confidence intervals and *p* values were estimated using the DBM/OR jackknife procedure applied separately to each pairwise comparison.

## Data Availability

The data presented in this study are available on request from the corresponding author. The data are not publicly available due to privacy and ethical restrictions.
